# Hepatorenal syndrome: an update

**DOI:** 10.1590/S1516-31802007000100010

**Published:** 2007-01-04

**Authors:** Tércio Genzini, Fábio César Miranda Torricelli

**Keywords:** Hepatorenal syndrome, Renal insufficiency, Liver cirrhosis, Portal hypertension, Vasoconstrictor agents, Síndrome hepatorrenal, Insuficiência renal, Cirrose hepática, Hipertensão portal, Agentes vasopressores

## Abstract

Hepatorenal syndrome (HRS) is the development of renal failure in patients with chronic previous liver disease, without clinical or laboratory evidence of previous kidney disease. It affects up to 18% of cirrhotic patients with ascites during the first year of follow-up, reaching 39% in five years and presenting a survival of about two weeks after its establishment. HRS diagnosis is based on clinical and laboratory data. The occurrence of this syndrome is related to the mechanism for ascites development, involving vasoconstriction, low renal perfusion, water and sodium retention, increased plasma volume, and consequent overflow at the splanchnic level. Renal vasoactive mediators like endothelin 1, thromboxane A2, and leukotrienes are also involved in the genesis of this syndrome, which culminates in functional renal insufficiency. The treatment of choice can be pharmacological or surgical, although liver transplantation is the only permanent and effective treatment, with a four-year survival rate of up to 60%. Liver function recovery is usually followed by renal failure reversion. Early diagnosis and timely therapeutics can increase life expectancy for these patients while they are waiting for liver transplantation as a definitive treatment.

## RESUMO

A síndrome hepatorrenal (SHR) é o desenvolvimento do quadro de insuficiência renal em pacientes com doença hepática crônica prévia sem evidências clínica ou laboratorial de nefropatia prévia. Atinge até 18% dos pacientes cirróticos com ascite em um ano, chegando a 39% em cinco anos, com uma sobrevida média em torno de duas semanas após estabelecido o quadro. O diagnóstico da SHR baseia-se em critérios clínicos e laboratoriais. Seu aparecimento está relacionado ao mecanismo de formação de ascite, que envolve vasoconstrição e hipofluxo renal, retenção de água e sódio, aumento do volume plasmático, e conseqüentemente hiperfluxo no território esplâncnico. Mediadores vasoativos renais e humorais, como a endotelina 1, tromboxano A2 e leucotrienos, estão ainda envolvidos na gênese desta síndrome que culmina com insuficiência renal funcional. O tratamento preconizado da SHR pode ser farmacológico ou cirúrgico, sendo o transplante de fígado o único efetivo e permanente, com sobrevida de até 60% em quatro anos. Após melhora da função hepática, geralmente há a reversão da insuficiência renal. O diagnóstico precoce e a rápida terapêutica podem ampliar a expectativa de vida destes hepatopatas enquanto se aguarda o transplante hepático para seu tratamento definitivo.

## INTRODUCTION

The word ascites comes from the Greek “askos”, which means “bag”, and it is defined as a liquid accumulation in the peritoneum, for which the main cause is liver cirrhosis.^1^ About 50% of the patients with compensated cirrhosis develop ascites within 10 years and, after its onset, 50% of these patients die within two to five years.^1^

A recent study by Gildea et al.^2^ revealed that hepatorenal syndrome (HRS) was the third most common cause of admission to hospital in the intensive care unit (ICU) among 420 patients with cirrhosis, and was only surpassed by upper digestive hemorrhage and encephalopathy. Mortality occurred within one to five years in 69% to 77% of all these patients. The risk factors identified were acute physiology, age, and chronic health evaluation (APACHE) III score of ≥ 90 (hazard ratio [HR], 2.2; 95% confidence interval [CI], 1.6 to 2.8; p < 0.0001), the use of pressors (HR, 2.5; 95% CI, 1.9 to 3.2; p < 0.0001), and jaundice (HR, 1.7; 95% CI, 1.4 to 2.2; p < 0.0001). Mortality occurred within 30 days in 92% of the cases presenting all three of these risk factors and in 11% of the cases without any of the above risk factors.

Hepatorenal syndrome (HRS) is defined as the development of renal insufficiency in patients with chronic liver disease, without clinical, laboratory or anatomical evidence that could justify its diagnosis. The most common causes of renal insufficiency in patients with liver disease are hypovolemia, drug nephrotoxicity, sepsis and glomerulonephritis. However, these causes must be ruled out for diagnosing HRS.^3^

HRS is considered to be a functional disease, since the renal histology remains normal, without morphological changes.^3^ It is characterized by oliguria, progressive azotemia, increased serum creatinine, hyponatremia and low levels of urinary sodium. This demonstrates the functional nature of the endangering of the kidneys. Sodium retention is generally the earliest alteration. This appears together with water retention and subsequent ascites formation.^4^

According to the International Ascites Club (IAC), two types of HRS can be defined: type I, or acute HRS, and type II, or chronic HRS. Type I has rapid evolution, generally between one and 14 days, with oliguria or enuresis. Such patients gene rally develop jaundice, encephalopathy and coagulation disorders, and renal insufficiency appears during hospitalization. The prognosis is poor, and the mean survival period is about two weeks.^5^ Type II or chronic HRS is characterized by progressive development of renal insufficiency, with increased creati-nine and urea within weeks or months, and generally presenting longer survival than in cases of type I HRS.

## BACKGROUND

One of the first descriptions of an association between kidney dysfunction and liver disease was given by Frerichs^6^ in the nineteenth century; he observed the presence of oliguria in patients with ascites. The absence of protein and the presence of low levels of sodium excretion in the urine of patients with HRS were reported by Hecker and Sherlock in 1956.^7^ The functional nature of this renal insufficiency was emphasized by Koppel et al.,^8^ who proved in the 1960s that kidneys obtained from dead patients with HRS could be transplanted into patients with renal insufficiency of different etiology, since these kidneys could recover their functional capacity after the transplantation. In the 1970s, Iwatsuki et al.^9^ showed that HRS patients recovered renal function after orthotopic liver transplantation, thus further highlighting the functional nature of the kidney dysfunction. Also in the 1960s and 1970s, papers by Schroeder et al.^10,11^ and Epstein et al.^12^ showed the role of renal vasoconstriction in the genesis of HRS. Schroeder focused on the hyperactivation of the renin-angiotensin-aldosterone system in its genesis, while Larsen et al.^13^ and Arroyo et al.^14^ suggested that action by the sympathetic nervous system was the causal factor for renal vasoconstriction in patients with cirrhosis. Boyer et al.^15^ showed that administration of non-hormonal anti-inflammatory drugs to cirrhotic patients with ascites provoked a considerable reduction in the renal blood flow and also the glomerular filtration rhythm (GFR). Subsequent studies attributed this behavior to reduction of the renal synthesis of vasodilatation prostaglandins, in which the vasoconstrictor systems are excessively activated for other reasons.

## EPIDEMIOLOGY

The cumulative likelihood of HRS occurrence in patients with cirrhosis with ascites can reach up to 18% over one year and up to 39% over five years, with mean survival period of about two weeks after the establishment of the syndrome.^5^ Retrospective studies have identified HRS in about 17% of the patients with ascites admitted to hospital and in more than 50% of deaths occurring among cirrhotic patients with liver failure.^16-18^ Some clinical and laboratory findings seem to indicate a greater tendency for HRS onset, among which clear sodium retention, low water depuration, dilutional hyponatremia, and hyposmolarity. There is also an association with absence of hepatomegaly and the presence of undernutrition and esophageal varices. On the other hand, liver function laboratory tests and the Child-Pugh scale have not been associated with the likelihood of HRS appearance.^5^

The epidemiological data available in the literature must be carefully analyzed, since many papers are discordant with the diagnostic criteria for HRS. Thus, some authors propose the inclusion of cases of renal insufficiency following digestive hemorrhage, bacterial infection or even nephrotoxic drug use.

## PHYSIOPATHOLOGY

In order to explain the mechanisms that lead to HRS, the theories for formation of the ascites that supplies the substrates and leads to renal vasoconstriction need to be understood. The first classical underfilling theory suggested that portal hypertension would favor lymph formation and that, whenever this production was greater than the lymphatic feedback, there would be ascites formation. This would then lead to hypovolemia and renal dysfunction with sodium and water retention.^19^

Concomitantly, several papers demonstrated that sodium and water retention and plasma volume expansion precede ascites formation.^20^ Thus, the most accepted theories for explaining renal vasoconstriction are:

1. “Overflow”, which suggests sodium retention as the initial mechanism, followed by a significant increase in volemia and ascites formation.^3^ According to this theory, the trigger factor for sodium retention would be sinusoidal hypertension, and expansion of arterial volemia associated with increased hydrostatic pressure in the splanchnic circulation would lead to the establishment of ascites through hyperflow;
2. Theory of peripheral arterial vasodilatation, proposed by Schrier et al. in 1988,^21^ in which cirrhosis and portal hypertension would cause progressive arterial vasodilatation, especially at splanchnic level, thus causing reduction of the effective volemia and subsequent activation of compensatory constrictive factors, such as the renin-angiotensin-aldosterone system, central nervous system, and antidiuretic hormone. The step following this consists of sodium and water retention. This arterial splanchnic vasodilatation is thought to play a fundamental role not only in the systemic hemodynamic alterations, but also in the increased hydrostatic pressure in the microcirculation, thus leading to overflow of the liquid from the intravascular space to the interstitial space.

All the theories for ascites formation partially explain the appearance of HRS since, one way or another, there is always support for renal vasoconstriction.

Among the factors involved in systemic arteriolar vasodilatation are nitric oxide (NO), glucagons, prostacyclins, potassium channels, endotoxins, cytokines and adeno-sine. NO is a vasodilator produced by the endothelium and smooth muscle cells of blood vessels. In patients with cirrhosis, the high plasma levels of nitrite and nitrate indicate increased NO production, which plays an important role in peripheral vasodilatation.^22^ The glucagon levels are also generally higher, and these cause desensitization of the mesenteric circulation to catecholamines and angiotensin II and, under pharmacological doses, they cause vasodilatation.^23^ Increased urinary excretion of prostacyclin metabolites in cirrhotic decompensated patients shows that their plasma levels are higher.^24^ The activation of potassium levels causes vasodilatation because of hyperpolarization of the smooth muscle cells of blood vessels, thus proving that the activation of these channels plays an important role in vasodilatation in cirrhotic patients.^25^ Endotoxinemia can also cause splanchnic vasodilatation, possibly mediated by cytokines and NO, and increased endotoxinemia in patients with decompensated cirrhosis is probably related to increased bacterial translocation and portosystemic shunts.^26^ Finally, adenosine plays a double mediation role, causing vasodilatation at the splanchnic level and renal vasoconstriction.^27^

The response to such systemic vasodilatation is the activation of mechanisms that lead to vasoconstriction aimed at maintaining arterial pressure, but these cause intense renal vasoconstriction that hampers the functioning of this organ.^17^ The vasoconstriction mediators are the sympathetic response, activation of the renin-angiotensin-aldosterone system, and increased vasopressin.^18^

Cases that progress towards HRS are characterized by considerably increased sinusoidal pressure and a certain degree of liver insufficiency that leads to excessive sympathetic activation. This exacerbated activation leads to large releases of catecholamines^28^ and this, together with the renin-angiotensin-aldosterone system (which is also stimulated in 50 to 80% of decompensated cirrhotic patients), leads to renal vasoconstriction, increased tubular reabsorption of sodium and water, reduced renal blood flow and glomerular filtration rate (GFR).^29^ Thus, HRS appears to be basically due to intense renal vasoconstriction, particularly in afferent renal arteries and arterioles. This vasoconstriction is mediated by interaction between several factors that have already been described, such as the reninangiotensin-aldosterone system, sympathetic nervous system, antidiuretic hormone, and endothelins (endothelial factors).^30^ This intense arterial and arteriolar vasoconstriction leads to low blood flow and GFR, while in the remaining circulation arteriolar vasodilatation prevails with low systemic resistance and arterial hypotension.

The presence of dilutional hyponatremia is generally mediated by increased plasma levels of the antidiuretic hormone (vasopressin), which leads to splanchnic and renal vasoconstriction and water retention, thus causing increased volemia and sodium dilution.^18^ Increased synthesis of renal and humoral vasoactive mediators is also part of HRS genesis. These mediators cause reductions in GFR, not through decreased renal blood flow, but through contraction of mesenchymal cells in the glomeruli, which leads to a reduction in the capillary coefficient of glomerular ultrafiltration (Kf) and a subsequent decrease in filtration fraction. Among these factors, endothelins,^31^ thromboxane A2,^17,32^ leukotrienes^17,33^ and isoprostanes^18,34^ can be highlighted.

Stimulation of the sympathetic nervous system causes a change in the self-regulatory curve for the renal blood flow, thereby making it more dependent on its perfusion pressure. Reduction in renal blood flow, caused by a fall in renal perfusion pressure, leads to renal functional insufficiency. For decompensated cirrhotic patients, several factors interact to decrease the renal blood flow, among which are a fall in mean arterial pressure,^35^ sympathetic hyperactivation and increased renal venous pressure or abdominal pressure after tense ascites.^36^

In patients with liver insufficiency, both plasma vasodilator levels and endogenous vasoconstrictors are increased. Concomitantly, the action of vasoconstrictors prevails through excessive sympathetic stimulation and activation of the renin-angiotensinaldosterone system. Moreover, increased intrarenal availability of vasoconstrictors such as thromboxane A2 and leukotrienes prevails over vasodilator substances, mainly represented by prostaglandins, prostacyclins and bradykinins.^17^ If the kidneys are subjected to such vasoconstriction conditions, consequent persistent ischemia lasting for several weeks or months can lead to tubular lesions, which are worsened when there is an association with nephrotoxic drugs.^27^

## DIAGNOSES

The HRS diagnostic criteria according to the IAC are presented in Table 1. The more important criteria must be present, while the less important criteria are not mandatory. It must be remembered that, although serum creatinine has high specificity for detecting low GFR, its sensitivity is low, probably because of reduced endogenous production of creatinine asso ciated with the protein undernutrition that is frequently observed in patients with cirrhosis. The sensitivity of 24-hour creatinine depuration for measuring GFR is greater than the sensitivity of serum creatinine, but it may give an overestimate and therefore careful urine 24-hour collection is needed. This is frequently impaired, since such patients are mostly oliguric.

**Table 1.** Diagnostic criteria for hepatorenal syndrome according to the International Ascites Club

Main criteria
Chronic or acute hepatic disease with severe hepatic insufficiency and portal hypertension. Low glomerular filtration rate, revealed by serum creatinine > 1.5 mg/dl or creatinine depuration < 40 ml/min in 24h. Absence of shock, bacterial infection, or recent or present treatment with nephrotoxic drugs. Absence of losses of gastrointestinal fluids (vomiting or diarrhea) or loss of renal fluids (weight loss > 500 g/day for several days in patients with ascites without peripheral edema or > 1000 g/day in patients with peripheral edema). Unsustainable improvement in renal function (serum creatinine decreased to 1.5 mg/dl or less; or creatinine depuration increased to 40 ml/min or more) after withdrawal of diuretics and expansion of the plasma volume with 1.5 l of isotonic saline solution or plasma expanders. Proteinuria < 500 mg/dl and absence of ultrasound evidence of obstructive uropathy or parenchymatous renal disease.
**Minor criteria (additional criteria)**	
Urine volume < 500 ml/day, urinary sodium < 10 mEq/l Urine osmolarity greater than plasma osmolarity. Urinary red cells < 50 per field Serum sodium concentration < 130 mEq/l

Adapted from Arroyo et al.^4^

Patients with cirrhosis are often exposed to a series of clinical situations that cause predisposition towards renal insufficiency that differs from HRS. Such situations, represented most frequently by digestive hemorrhage and bacterial infections, must be ruled out from the HRS diagnosis, since they can cause decreased arterial pressure with bad tissue perfusion (shock) and also acute tubular necrosis. It is important to stress that HRS patients may present acute tubular necrosis because of intense vasoconstriction that leads to ischemia. About one third of cirrhotic patients with spontaneous bacterial peritonitis develop renal insufficiency^37^ and one third of such cases reverse this condition after curing the peritonitis with appropriate antibiotic therapy.

Several drugs are especially nephrotoxic in cirrhotic patients. These include non-hormonal anti-inflammatory drugs, which inhibit the synthesis of renal prostaglandins through their vasodilator action; aminoglycosides, which increase the predisposition towards acute tubular necrosis; and diuretics, which can cause renal insufficiency, particularly in patients without peripheral edema. In other situations that lead to renal insufficiency, such as depletion of intravascular volume through significant losses of gastrointestinal fluids (in cases of vomiting or diarrhea, for instance), there is a reduction in renal perfusion and low GFR, and these situations are reversed after restoring the intravascular volume by means of plasma expansion factors. For HRS patients, there is no improvement in renal function following plasma expansion, and this is one of the main criteria for diagnosing HRS. A therapeutic test using rapid infusion of 1.5 l of isotonic saline solution simultaneously with suspension of diuretics must be performed. Improvement in renal function is shown by a decrease in serum creatinine to less than or equal to 1.5 mg/dl or by an increase in creatinine depuration to greater than or equal to 40 ml/min,^4^ when HRS is not present. Moreover, absence of significant glomerular or tubular lesion generally contributes towards absence of proteinuria. There are generally additional criteria, but these are not fundamental for the diagnosis. HRS patients present urinary sodium less than 10 mEq/l, since renal tubular function is preserved and the kidneys do not lose their capacity for so dium reabsorption. In some cases, urinary sodi um may be present at a concentration of more than 20 mEq/l, with the presence or absence of acute tubular necrosis.^38^ Urinary osmolarity is generally greater than plasma osmolarity and in most cases there is no hematuria, which is an indication that there is no glomerular lesion.

Damage to the renal capacity for excreting free water is an almost universal characteristic of HRS. This is the triggering factor for dilutional hyponatremia, which can be severe in some cases.

Type 1 HRS is an acute form that is generally associated with the most severe cases of hepatic disease. It is more frequent in cases of alcoholic hepatitis, or after acute decompensation of hepatic cirrhosis in patients who generally present significant coagulation disorders. It is characterized by rapidly progressive reduction in renal function, shown by doubling of the serum creatinine levels to more than 2.5 mg/dl or by 50% reduction in the initial 24-hour creatinine depuration to less than 20 ml/min within two weeks.^18^

In such cases, renal function recovers spontaneously with the improvement in hepatic function. The prognosis is poor, with mortality of about 80% within two weeks.^18^

In type 2 HRS, functional renal insufficiency does not progress rapidly. It generally affects patients with voluminous ascites who are resistant to diuretics. The serum creatinine levels are usually greater than 1.5 mg/dl and creatinine depuration is less than 40 ml/min. It may change to type 1, and in such cases its prognosis is also poor.^18^

## TREATMENT

Liver transplantation is the most effective and definitive treatment for HRS. However, HRS patients present greater morbidity and mortality, with lower survival rates. Gonwa et al.^39^ showed that there was no difference in perioperative (90-day) mortality. Oneand two-year actuarial survival rates in the non-HRS patients were 87.2% and 82.1%, respectively. The actuarial one- and two-year survival rate for the HRS patients was 76.6% (p > 0.005) The improvement in liver function following liver transplantation reverses the condition of functional renal insufficiency.

If liver transplantation is impossible, the approach for HRS patients includes firstly volemia expansion and then confirmation of the diagnosis, if there was no continuous improvement in renal function. Although the IAC recommends that such volemic expansion must be done with 1.5 l of isotonic saline solution, many groups prefer to use colloidal substances, particularly solutions containing human albumin.^18,37,40^

The use of paracentesis, especially in cases of tense ascites, can improve renal function through improving the renal blood flow, since there is a reduction in renal venous pressure. Large-volume paracentesis is when there is drainage of more than five liters of ascites fluid. This is associated with circulatory problems in up to 20% of such cases, which are represented by development or worsening of hyponatremia and increased circulatory catecholamines and renin activity.^1^ The most significant alterations occur between 24 and 48 hours after large-volume paracentesis and these are associated with greater risk of renal insufficiency and mortality within 30 days. Replacement of 8 g of albumin per liter of ascites fluid drained is recommended, in order to avoid severe circulatory problems, but this is not needed in cases of paracentesis of less than five liters.^41^

The improvement in renal function following paracentesis or volemic expansion is transitory.^40^ More sustainable improvement in renal function can be obtained with the use of peripheral and splanchnic vasoconstrictive drugs, which increase the perfusion pressure and overcome the effects of renal vasoconstriction. It is important to stress that there are no drugs that act only at the splanchnic level. Therefore, some of these vasoconstrictors may worsen HRS since they would also contribute towards intensifying the renal vasoconstriction.^18^

Low doses of dopamine have a renal vasodilator effect, which increases diuresis in about 5% of the cases. Thus, some authors recommend dopamine use for 12 hours and stopping it if there is no response seen through increased diuresis.^18,42^ This effect is temporary and prolonged dopamine use must be avoided, since it stimulates catabolism.

Ornipressin and vasopressin likewise provoke vasoconstriction, especially at the splanchnic level. They reduce the renin and angiotensin plasma levels and increase the flow and pressure for renal perfusion. When administered with plasma expanders, particularly human albumin, good results are obtained, with increased mean arterial pressure and improvement in renal function.^17,18,43-45^ There have also been reports of good results using associations of ornipressin and dopamine.^43^

Terlipressin, also known as triglycyllysine vasopressin, is similar to vasopressin and can improve renal function in cirrhotic HRS patients. Because of its vasoconstrictor effect at the splanchnic level and subsequent hemodynamic improvement, suppression of renal vasoconstrictor activity may occur, with improvement in kidney function and increased potassium excretion. Moreover, the possible direct effect of tubular potassium excretion may also contribute towards good results in treating hyperkalemia associated with HRS.^46^

Combined administration of midodrine and octreotide seems to improve survival in HRS cases.^47^ Midodrine is a sympathomimetic drug, while octreotide is similar to prolonged-action somatostatin, and long-term administration of octreotide increases renal blood flow.^48^

Other drugs like misoprostol, which is a synthetic drug similar to prostaglandin E1,^49,50^ endothelin antagonists,^51^ which inhibit the powerful vasoconstriction of endothelin, and n-acetylcysteine,^52^ may also be useful for treating HRS.^18^

Peritoneal venous shunts avoid excessive increases in abdominal pressure, maintain volemic expansions and stimulate, through distension of the right atrium, increased production of atrial natriuretic factor, and these have a positive effect on HRS treatment. However, the high complication rate from these shunts, which is related to coagulation disorders and valve obstruction (observed up to 40% of cases^1^), plus the fact that they can facilitate ascites fluid infection, limits their use in practice to patients who are not selected for liver transplantation. The prosthesis most used for peritoneal venous shunts is the Le Veen valve,^1^ which moves when the pressure gradient between the abdomen and the right atrium is greater than or equal to 3 cmH_2_O.

Maintained volemic expansion and simultaneous improvement in sinusoidal hypertension can be achieved with portosystemic shunts. The severity of such patients’ conditions only allows the use of a transjugular intrahepatic portosystemic shunt (TIPS), since it is less invasive than surgical shunts.^53^ Patients with low hepatic reserves, as shown through total bilirubin volume greater than 3 mg/dl, creatinine greater than 2 mg/dl and prothrombin time greater than 20 seconds, present mortality greater than 90% within three months after performing TIPS.^1^ Further-more, the prosthesis obstruction index may reach 75% and encephalopathy can occur in about 30% of the cases. Thus, the use of TIPS is presently accepted as a temporary treatment that precedes liver transplantation.^1^

Renal dialysis support can only be used in cases in which there is a real possibility of reestablishing liver function or in which liver transplantation has been selected. Continuous hemofiltration is a better-tolerated way of providing renal support, since intermittent hemofiltration may cause hemodynamic instability and clinic worsening in some patients.^35^

It is known that the levels of many endogenous molecules found in intracellular sections become pathologically elevated when there is a reduction in acute or chronic hepatic albumin synthesis. Among such molecules are bilirubins, albumin, aromatic amino acids, biliary acids, endogenous benzodiazepines, mercaptans, nitric oxide, prostacyclins, and triptophans. Furthermore, many potentially toxic drugs like phenytoin are related to albumin.^54^

The capacity of albumin for binding to several molecules makes it a potential dialysis agent for acute and chronic patients with liver disease. This is the matter that has most recently been addressed with regard to liver dialysis.

The molecular absorbent and recirculating system (MARS) is the most frequently used albumin dialysis system. Its reported effects are improvement of encephalopathy, reduction of intracranial pressure, reduction of creatinine and ammonium serum levels, and increased Factor VII, albumin and branched-chain amino acids. Recent studies have reported MARS use for HRS treatment, showing that, in comparison with hemodialysis, MARS is better with regard to sodium, creatinine and bilirubin levels and prothrombin time, while it presents similar results for blood albumin, diuresis and mean arterial pressure. The mean survival of patients is also significantly greater with MARS than with hemodialysis or hemofiltration.^48,55^

In addition to peritoneal venous shunt and TIPS, sympathectomy has also been proposed as a surgical option for HRS treatment. In a small report on eight patients, Solis-Herruzo^56^ suggested that sympathetic block might improve renal function in cirrhotics with HRS, particularly among those with more impaired GFR. In this study, five patients presented basal GFR below 25 ml/min, and in these cases sympathetic block induced a significant increase in GFR, osmolal clearance, urinary sodium excretion, fractional excretion of filtered sodium and effective renal plasma flow and a decrease in plasma rennin activity. All these measures are palliative and used while awaiting improvement of liver function through regeneration resulting from transplantation.

Tables 2, 3 and 4^57^ show the therapeutic procedures for HRS with their respective levels of evidence and grades of recommendation.

**Table 2.** Therapeutic procedures for hepatorenal syndrome with respective level of evidence and grade of recommendation

References	Treatment	Level of evidence	Grade of recommendation
Gonwa TA, et al.^39^	Liver transplant	III	A
Gines A, et al.^41^	Paracentesis with (> 5 l) or without albumin	III	A
Gulberg V, et al.^43^ and Hadengue A, et al.^44^	Ornipressin/terlipressin	III	A
Angeli P, et al.^47^	Midodrine/octreotide	III	A
Bennett WM, et al.^42^	Dopamine	IV	B
Gines A, et al.^41^	Albumin	III	B
Roberts LR, et al.^40^	Isotonic saline solution	IV	B
Fevery J, et al.^49^ and Gines A, et al.^50^	Prostaglandin analogues - misoprostol	IV	B
Soper CP, et al.^51^	Endothelin antagonist	IV	B
Holt S, et al.^52^	N-acetylcysteine	IV	B
Brensing KA, et al.^53^	Transjugular intrahepatic portosystemic shunt	IV	B
Moore K^35^	Dialysis	IV	B
Mitzner SR, et al.^55^	Molecular absorbent and recirculating system	III	B
Solis-Herruzo JA, et al.^56^	Lumbar sympathetic	VI	B

**Table 3.** Levels of evidence for therapy and prevention

I	Systematic review with meta-analysis
II	Megatrial (> 1000 patients)
III	Randomized clinical trial (< 1000 patients)
IV	Cohort (not randomized)
V	Case-control study
VI	Case series (without control group)
VII	Specialist opinion

**Table 4.** Grades of recommendation for decision on method^57^

A	Evidence is strong enough to indicate method
B	Evidence has not been established
C	Evidence is strong enough to contraindicate method

## CONCLUSION

The etiopathogenesis of HRS is closely related to the mechanisms for the establishment of ascites. Understanding these mechanisms, as the factors leading to HRS itself, facilitates diagnosis and subsequent therapy for HRS. However, the prognosis remains limited, even with the new vasopressors and therapeutic schemes. Recovery of renal function occurs after liver improvement, through hepatocyte regeneration processes or liver transplantation.

## References

[1] Sandhu BS, Sanyal AJ (2005). Management of ascites in cirrhosis. Clin Liver Dis.

[2] Gildea TR, Cook WC, Nelson DR (2004). Predictors of long-term mortality in patients with cirrhosis of the liver admitted to a medical ICU. Chest.

[3] Gattoni A, Marotta F, Vangieri B, Pisani G, Cristiano F (2004). Hepatorenal syndrome. Clin Ter.

[4] Arroyo V, Gines P, Gerbes AL (1996). Definition and diagnostic criteria of refractory ascites and hepatorenal syndrome in cirrhosis. International Ascites Club. Hepatology.

[5] Gines A, Escorsell A, Gines P (1993). Incidence, predictive factors, and prognosis of the hepatorenal syndrome in cirrhosis with ascites. Gastroenterology.

[6] Frerichs T (1877). Tratado práctico de las enfermedades del hígado, de los vasos hepáticos y de las vías biliares.

[7] Hecker R, Sherlock S (1956). Electrolyte and circulatory changes in terminal liver failure. Lancet.

[8] Koppel MH, Coburn JM, Mims MM, Goldstein H, Boyle JD, Rubini ME (1969). Transplantation of cadaveric kidneys from patients with hepatorenal syndrome. Evidence for the functionalnature of renal failure in advanced liver disease. N Engl J Med.

[9] Iwatsuki S, Popovtzer MM, Corman JL (1973). Recovery from “hepatorenal syndrome” after orthotopic liver transplantation. N Eng J Med.

[10] Schroeder ET, Shear L, Sancetta SM, Gabuzda GJ (1967). Renal failure in patients with cirrhosis of the liver. 3. Evaluation of intrarenal blood flow by para-aminohippurate extraction and response to angiotensin. Am J Med.

[11] Schroeder ET, Eich RH, Smulyan H, Gould AB, Gabuzda GJ (1970). Plasma renin level in hepatic cirrhosis. Relaton to functional renal failure. Am J Med.

[12] Epstein M, Berk DP, Hollenberg NK (1970). Renal failure in the patient with cirrhosis. The role of active vasoconstriction. Am J Med.

[13] Ring-Larsen H, Hesse B, Henriksen JH, Christensen NJ (1982). Sympathetic nervous activity and renal and systemic hemodynamics in cirrhosis: plasma norepinephrine concentration, hepatic extraction, and renal release. Hepatology.

[14] Arroyo V, Planas R, Gaya J (1983). Sympathetic nervous activity, renin-angiotensin system and renal excretion of prostaglandin E2 in cirrhosis. Relationship to functional renal failure and sodium and water excretion. Eur J Clin Invest.

[15] Boyer TD, Zia P, Reynolds TB (1979). Effect of indomethacin and prostaglandin A1 on renal function and plasma renin activity in alcoholic liver disease. Gastroenterology.

[16] Suzuki H, Stanley AJ (2001). Current management and novel therapeutic strategies for refractory ascites and hepatorenal syndrome. QJM.

[17] Gentilini P, Vizzuti F, Gentilini A, La Villa G (2001). Ascites and hepatorenal syndrome. Eur J Gastroenterol Hepatol.

[18] Dagher L, Moore K (2001). The hepatorenal syndrome. Gut.

[19] Witte CL, Witte MH, Dumont AE (1980). Lymph imbalance in the genesis and perpetuation of the ascites syndrome in hepatic cirrhosis. Gastroenterology.

[20] Levy M, Wexler MJ (1978). Renal sodium retention and ascites formation in dogs with experimental cirrhosis but without portal hypertension or increased splanchnic vascular capacity. J Lab Clin Med.

[21] Schrier RW, Arroyo V, Bernardi M, Epstein M, Henriksen JH, Rodes J (1988). Peripheral arterial vasodilatation hypothesis: a proposal for the initiation of renal sodium and water retention in cirrhosis. Hepatology.

[22] Guarner C, Soriano G, Tomas A (1993). Increased serum nitrite and nitrate levels in patients with cirrhosis: relationship to endotoxemia. Hepatology.

[23] Pak JM, Lee SS (1994). Glucagon in portal hypertension. J Hepatol.

[24] Guarner F, Guarner C, Prieto J (1986). Increased synthesis of systemic prostacyclin in cirrhotic patients. Gastroenterology.

[25] Moreau R, Lebrec D (1995). Endogenous factors involved in the control of arterial tone in cirrhosis. J Hepatol.

[26] Lumsden AB, Henderson JM, Kutner MH (1988). Endotoxin levels measured by a chromogenic assay in portal, hepatic and peripheral venous blood in patients with cirrhosis. Hepatology.

[27] Lee SS, Chilton EL, Pak JM (1992). Adenosine receptor blockade reduces splanchnic hyperemia in cirrhotic rats. Hepatology.

[28] Gaudin C, Braillon A, Poo JL, Moreau R, Hadengue A, Lebrec D (1991). Regional sympathetic activity, severity of liver disease and hemodynamics in patients with cirrhosis. J Hepatol.

[29] Henriksen JH, Ring-Larsen H (1994). Hepatorenal disorders: role of the sympathetic nervous system. Semin Liver Dis.

[30] Schrier RW, Neiederberger M, Weigert A, Gines P (1994). Peripheral arterial vasodilatation: determinant of functional spectrum of cirrhosis. Semin Liver Dis.

[31] Gentilini P (1999). Hepatorenal syndrome and ascites--an introduction. Liver.

[32] Zipser RD, Radvan GH, Kronborg IJ, Duke R, Little TE (1983). Uri-nary thromboxane B2 and prostaglandin E2 in the hepatorenal syndrome: evidence for increased vasoconstrictor and decreased vasodilator factors. Gastroenterology.

[33] Huber M, Kastner S, Scholmerich J, Gerok W, Keppler D (1989). Analysis of cysteinyl leukotrienes in human urine: enhanced excretion in patients with liver cirrhosis and hepatorenal syndrome. Eur J Clin Invest.

[34] Morrow JD, Moore KP, Awad JA (1993). Marked overproduction of non-cyclooxygenase derived prostanoids (F2-isoprostanes) in the hepatorenal syndrome. J Lipid Mediat.

[35] Moore K (1997). The hepatorenal syndrome. Clin Sci (Lond).

[36] Mullane JF, Gliedman ML (1966). Elevation of the pressure of the abdominal inferior vena cava as a cause of a hepatorenal syndrome in cirrhosis. Surgery.

[37] Follo A, Llovet JM, Navasa M (1994). Renal impairment after spontaneous bacterial peritonitis in cirrhosis: incidence, clinical course, predictive factors and prognosis. Hepatology.

[38] Dudley FJ, Kanel GC, Wood LJ, Reynolds TB (1986). Hepatorenal syndrome without avid sodium retention. Hepatology.

[39] Gonwa TA, Morris CA, Goldstein RM, Husberg BS, Klintmalm GB (1991). Long-term survival and renal function following liver transplantation in patients with and without hepatorenal syndrome--experience in 300 patients. Transplantation.

[40] Roberts LR, Kamath PS (1996). Ascites and hepatorenal syndrome: pathophysiology and management. Mayo Clin Proc.

[41] Gines A, Fernandez-Esparrach G, Monescillo A (1996). Randomized trial comparing albumin, dextran 70, and polygeline in cirrhotic patients with ascites treated by paracentesis. Gastroenterology.

[42] Bennett WM, Keeffe E, Melnyk C, Mahler D, Rosch J, Porter GA (1975). Response to dopamine hydrochloride in the hepatorenal syndrome. Arch Intern Med.

[43] Gulberg V, Bilzer M, Gerbes AL (1999). Long-term therapy and retreatment of hepatorenal syndrome type 1 with ornipressin and dopamine. Hepatology.

[44] Hadengue A, Gadano A, Moreau R (1998). Beneficial effects of the 2-day administration of terlipressin in patients with cirrhosis and hepatorenal syndrome. J Hepatol.

[45] Uriz J, Gines P, Cardenas A (2000). Terlipressin plus albumin infusion: an effective and safe therapy of hepatorenal syndrome. J Hepatol.

[46] Kalambokis G, Milionis H, Elisaf M, Tsianos EV (2005). Terlipressin avoids hemodialysis in the treatment of refractory hyperkalemia associated with renal dysfunction in cirrhosis. Am J Med.

[47] Angeli P, Volpin R, Gerunda G (1999). Reversal of type 1 hepatorenal syndrome with the administration of midodrine and octreotide. Hepatology.

[48] Angeli P, Volpin R, Piovan D (1998). Acute effects of the oral administration of midodrine, an alpha-adrenergic agonist, on renal hemodynamics and renal function in cirrhotic patients with ascites. Hepatology.

[49] Fevery J, Van Cutsem E, Nevens F, Van Steenbergen W, Verberckmoes R, De Groote J (1990). Reversal of hepatorenal syndrome in four patients by peroral misoprostol (prostaglandin E1 analogue) and albumin administration. J Hepatol.

[50] Gines A, Salmeron JM, Gines P (1993). Oral misoprostol or intravenous prostaglandin E2 do not improve renal function in patients with cirrhosis and ascites with hyponatremia or renal failure. J Hepatol.

[51] Soper CP, Latif AB, Bending MR (1996). Amelioration of hepatorenal syndrome with selective endothelin-A antagonist. Lancet.

[52] Holt S, Goodier D, Marley R (1999). Improvement of renal function in hepatorenal syndrome with N-acetylcysteine. Lancet.

[53] Brensing KA, Textor J, Perz J (2000). Long term outcome after transjugular intrahepatic portosystemic stent-shunt in non-transplant cirrhotics with hepatorenal syndrome: a phase II study. Gut.

[54] Barshes NR, Gay AN, Williams B, Patel AJ, Awad SS (2005). Support for the acutely failing liver: a comprehensive review of historic and contemporary strategies. J Am Coll Surg.

[55] Mitzner SR, Stange J, Klammt S (2000). Improvement of hepatorenal syndrome with extracorporeal albumin dialysis MARS: results of a prospective, randomized, controlled clinical trial. Liver Transpl.

[56] Solis-Herruzo JA, Duran A, Favela V (1987). Effects of lumbar sympathetic block on kidney function in cirrhotic patients with hepatorenal syndrome. J Hepatol.

[57] Atallah AN, Trevisani VFM, Valente O, Borges DR, Rothschild HA (2003). Princípios para tomadas de decisões terapêuticas com base em evidências científicas. Atualização Terapêutica.

